# Short‐Forms of the Brazilian Children's Experiences of Dental Anxiety Measure (CEDAM): Development and Initial Evaluation

**DOI:** 10.1111/ipd.13326

**Published:** 2025-06-12

**Authors:** Lucas Daniel da Silva Faria, Brenda Soares Ribeiro, Julia Henriques Lamarca dos Santos, Taís de Souza Barbosa

**Affiliations:** ^1^ Department of Social and Pediatric Dentistry Institute of Science and Technology—Campus of São José dos Campos, São Paulo State University (Unesp) São José dos Campos São Paulo Brazil; ^2^ Postgraduate Program in Oral Health Applied Sciences Institute of Science and Technology—Campus of São José dos Campos, São Paulo State University (Unesp) São José dos Campos São Paulo Brazil; ^3^ Postgraduate Program in Dentistry Faculty of Dentistry, Federal University of Juiz de Fora (UFJF) Governador Valadares Minas Gerais Brazil

**Keywords:** child, dental anxiety, reliability and validity, surveys and questionnaires

## Abstract

**Background:**

Dental anxiety can be measured by self‐report instruments. The Children's Experiences of Dental Anxiety Measure (CEDAM) is a 14‐item measure of dental anxiety. A short form would be useful to reduce application time and financial costs of data collection.

**Aims:**

To develop the short‐forms of the Brazilian CEDAM to facilitate its use and to evaluate their psychometric properties using item impact and regression methods.

**Design:**

The sample consisted of 80 9–12‐year‐old schoolchildren. The item impact method selected the eight items with the highest impact score and multiplied the frequency of individuals by the mean score of the item. The regression method considered the total score as the dependent variable and the individual item scores as independent variables. Content, Criterion, Construct Validity, and Reliability were tested.

**Results:**

The methods identified 4 common questions. Short‐forms showed a floor effect, and the impact method had a ceiling effect (Content Validity). Short‐form scores positively correlated with the CEDAM (Criterion Validity) and the Children's Fear Survey Schedule‐Dental Subscale (CFSS‐DS) scores (Correlational Construct Validity). High fearful children (CFSS‐DS ≥ 32) had higher short‐form scores than their counterparts (Discriminant Construct Validity). Short‐forms showed to be reliable (Cronbach's *α* > 0.80).

**Conclusions:**

Short‐forms of the Brazilian CEDAM demonstrated satisfactory validity and reliability.

**Summary**

- Why this paper is important to pediatric dentists
  ○ The Brazilian short‐forms of the CEDAM would be useful to reduce the time of application and financial costs of data collection.
  ○ The risk of total and item non‐response is potentially reduced using short‐forms.
  ○ These short‐form would be useful tools for clinicians to evaluate the presence and severity of dental anxiety self‐reported by children.

## 1 Introduction

Dental anxiety denotes a state of apprehension that something dreadful related to dental treatment is going to happen, and it is coupled with a sense of losing control [1]. The multifactorial etiology of children's dental anxiety involves previous painful dental experience and dental fear of parents, which can compromises their adherence to dental treatment [2]. Dental anxiety can be measured by self‐report instruments [3]. In recent years, different measures have been developed, with both potential and limitations [4]. The Children's Experiences of Dental Anxiety Measure (CEDAM) is a 14‐item self‐report questionnaire, developed in English, which assesses feelings and behavioral, cognitive, and physical symptoms related to dental anxiety in 9–16‐year‐old children [3]. The instrument was developed in accordance with the Five Areas theoretical model of Cognitive Behavioral Therapy (CBT), which is a short‐term, problem‐focused psychosocial intervention [5]. The Five Areas approach is a more pragmatic and accessible model of assessment using CBT, whose aim is to communicate the fundamental principles of CBT and the main clinical interventions in clear language. Among the five areas the model covers are: life situation, relationships and practical problems; altered thinking; altered emotions; altered physical feelings and/or symptoms; altered behaviors or activity levels [5].

CEDAM has good psychometric properties for use in research, clinical settings, and health care evaluations [3, 6]. The advantages of the CEDAM, over other available measures, are assessing experiences that anxious children specifically identify as central to their anxiety and using accessible language and concepts appropriate for this group. Nevertheless, few clinicians use validated scales when assessing patients [7]. Most dentists only consider their self‐perception to evaluate the level of anxiety in children [8], which makes it difficult to know the real needs of the child patient. Limitations in the use of subjective measures mainly refer to the child's difficulty in completing the questionnaire, time limitations, and inexperience in applying questionnaires [6]. Thus, the measurement of dental anxiety in clinical practice requires brief, direct instruments [6] and language accessible to children [9].

In the literature, there is no construction protocol for short forms of questionnaires, nor is there a minimum number of questions needed to be used. But there is a diversity of methodologies that can be considered to create instruments, and short forms with at least six questions have demonstrated satisfactory measurement properties [10, 11]. The use of statistical approaches alone to develop short forms does not seem to be the best option [10], and a combination of theoretical considerations and statistical techniques has been recommended [12]. In the regression method, items were ranked by their contribution to the coefficient of variance (*R* ^2^) [13]. The item impact method is a good option when the aim is to prioritize the most important items for the patient [14]. This method would be suitable for CEDAM as it is a child‐centered measure. However, considering that the CEDAM was developed based on a theory, it is important that this theoretical framework is also used to create the short form [12].

In clinical practice, the use of short‐forms is an advantage. Porrit et al. [13] developed the 8‐item versions of the CEDAM in English in a sample of schoolchildren and patients aged 9 to 16 years, using the impact and regression methods. The short‐forms showed good measurement properties and are recommended for use in clinical routine due to their ease and speed of administration [13]. The Brazilian version of the original CEDAM has already been developed and validated for schoolchildren aged 9 to 12 years [2]. The Brazilian CEDAM performed well as a discriminative measure, being able to distinguish between higher fearful children and low fearful ones, and showed almost perfect internal consistency (Cronbach's *α* = 0.93). Nevertheless, the use of the measure in clinical settings may be limited by the additional scoring. A short‐form of the Brazilian CEDAM, which does not require additional scores [14], would promote its use in clinical practice. Moreover, it would be useful to reduce the time of application and financial costs of data collection. Moreover, the risk of total and item non‐response are potentially reduced using short‐forms.

Considering that the English short‐forms did not represent the items with greater impact and more relevance for the Brazilian population and the fact that dental anxiety can be mediated by other factors, such as personal, social, cultural, and environmental variables, the present study aimed (1) to develop the short‐form of the Brazilian version of the CEDAM and (2) to evaluate the psychometric properties of the 8‐item versions using two methods, impact and regression.

## 2 Material and Methods

### 2.1 Ethical Aspects

The cross‐sectional study was approved by the Human Research Ethics Committee of the Federal University of Juiz de Fora—UFJF (Protocol number: CAAE: 55005421.7.0000.5147) and written informed assent from children and consent from parents were obtained prior to starting data collection.

### 2.2 Sample

Data were obtained from the validity and reliability study of the Brazilian version of 14‐item CEDAM [2]. The sample was composed of 80 children, aged 9 to 12 years, recruited from State School Coronel Camilo Soares, Ubá, MG, Brazil, during the period of September 2022 to April 2023.

The inclusion criteria considered were the acceptance of the Informed Consent and Assent Forms, the participation in all phases of the study, and the completion of the questionnaires. Children who did not collaborate with data collection and parents who did not fill out any essential data, such as telephone number, were excluded.

The sample size was calculated based on the CEDAM score obtained from the validity study of Porritt et al. [3] Considering a standard deviation of 3.73 (the higher value between nine to twelve‐year‐old children), a margin of error of 2%, a confidence level of 95%, and a sample loss of 20% [15]; the required sample size was defined as 20 children for each age group; that is, 80 children aged between nine and twelve years old [2].

### 2.3 CEDAM

CEDAM measures child's self‐report of thoughts, behaviors, physical symptoms, and feelings related to dental anxiety. The questionnaire consists of 14 items with 3 possible response options, ranging from 1 to 3 points (e.g., 3. When I next visit the dentist… I will let the dentist look in my mouth [score = 1]; I will try to stop the dentist looking in my mouth a bit [score = 2]; or I will not let the dentist look in my mouth [score = 3]) [3]. The total score (14 to 42) is obtained by the sum of scores of all questions; higher scores indicate a higher level of dental anxiety. While the CEDAM‐14 requires transformation of scores when it is used in research to detect change, the short forms do not require transformation of raw scores and therefore overcome this potential barrier in clinical practice [2].

### 2.4 Development of the Short‐Forms

The 8‐item short‐forms were developed using item impact and regression methods [16, 17]. Each item impact score was calculated by multiplying the frequency of individuals who reported greater anxiety (score 3) by the item's mean value of CEDAM [18]. The 8 highest ranked items were included in the CEDAM‐ISF:8.

In the regression method, the dependent variable was the CEDAM overall score, while all the items were the independent variables. A multiple regression analysis with the forward stepwise method was performed. The 8 items with the highest contribution to the coefficient of variance (*R* ^2^) were included in the CEDAM‐RSF:8.

### 2.5 Evaluation and Comparison of the Short‐Forms

The psychometric properties of the short‐forms (CEDAM‐ISF:8 and CEDAM‐RSF:8) were assessed using the data from the validity and reliability study for the Brazilian CEDAM‐14 [2]. The responses of 80 nine‐to eleven‐year‐old school children were used to test the validity and reliability of the short‐forms. Overall scores were obtained by summing the response codes to their items.

In the previous study [2], children were clinically examined at school for dental caries, dental trauma, gingivitis, and fluorosis by one trained examiner. The dental caries were evaluated by dmft and DMFT (decayed, missing and filled teeth in the primary and permanent dentition, respectively) [19]. The presence or absence of dental trauma and gingivitis was classified as “yes” or “no” respectively. The Dean's index criteria were used to categorize the level of fluorosis as: 0, normal; 1, questionable; 2, very mild; 3, mild; 4, moderate; and 5, severe [20]. In the present study, these data were used to characterize the evaluated sample.

For testing construct validity, the data obtained from the Brazilian version of the Children's Fear Survey Schedule‐Dental Subscale (CFSS‐DS) [19] was used. The questionnaire showed to be a valid and reliable tool for the assessment of dental fear in children aged 4–12 years (Cronbach's *α* = 0.77–0.88; Weighted Kappa = 0.76) [21]. At the school, the children self‐applied the 15 items of CFSS‐DS, with responses options in Likert scale of five points, ranging from “not afraid” (score 1) to “very afraid” (score 5) [22, 23]. The sum of the total score of CFSS‐DS can vary between 15 to 75 points [21]. Based on previous studies, the following cutoff scores and classification was used “low fearful children” (score < 32) and “high fearful children” (≥ 32) [24, 25].

### 2.6 Statistical Analysis

The data was analyzed using JAMOVI 2.3.17 software, with a significance level of *α* = 0.05. The overall scores of the short‐forms were summed by the response codes for all the questions. Data distribution was checked using the Kolmogorov–Smirnov normality test. Content validity examines the extent to which the concepts of interest are comprehensively represented by the items in the questionnaire [26]. Content validity was determined by the floor and ceiling effects for both versions. Moreover, the comparison between the means of short‐forms was performed using Mann–Whitney test. Criterion validity establishes the validity of a measurement instrument by comparing it with external criteria [27]. Criterion validity was assessed by correlating the CEDAM score with the scores of the short‐forms, using Spearman's correlation test. Construct validity refers to the degree to which an instrument consistently relates to other similar measurements derived from the same theory and concepts [28]. For correlational construct validity analysis, Spearman's correlation test was used to associate the scores of the short‐forms with the CFSS‐DS overall scores. The magnitude of the correlation considered the following values: 0.9 to 1.0 (“very” strong correlation); 0.7 to 0.89 (“strong”); 0.4 to 0.69 (“moderate”); 0.2 to 0.39 (“weak”); a to 0.19 (“no or very weak”). For discriminant construct validity, the means of the short versions were compared according to the CFSS‐DS categories (< 32, low fearful; ≥ 32, high fearful), using the Mann–Whitney test. Internal consistency indicates whether the items of a questionnaire measure the same phenomenon [28] and was determined by calculating Cronbach's alpha coefficient, alpha if item deleted and corrected item‐total correlation. Internal consistency can be classified as: “almost perfect” (> 0.80), “substantial” (0.61 to 0.80); “moderate” (0.41 to 0.60); “fair” (0.21 to 0.40), “poor” (< 0.21) [29]. Values higher than 0.70 was considered satisfactory.

## 3 Results

Table 1 shows the characteristics of the evaluated sample. While the majority of children were caries‐free (66.3%) (*p* = 0.0245), the experience of dental caries in primary and permanent dentitions was observed in 57.7% (*p* = 0.0045) and 27.6% (< 0.0001), respectively. The presence of dental trauma, fluorosis and gingivitis was observed in less than 10% of the sample.

**TABLE 1:** Descriptive analysis: characteristics of the evaluated sample (*n* = 80).

Characteristics	Categories	*n* (%)	*p*
Sex (*n* [%])	Female	39 (48.8)	0.9636*
	Male	41 (51.3)	
Age	Mean (SD)	40 (50.0)	—
Presence of decayed tooth	Yes	27 (33.8)	**0.0245** *
	No	**53 (66.3)**	
dmft score	0	25 (31.3)	**0.0045** **
	1–2	**26 (32.5)**	
	3–4	14 (17.5)	
	≥ 5	6 (7.5)	
	Mean (SD)	1.8 (2.0)	
DMFT score	0	**58 (72.5)**	< **0.0001** **
	1–2	17 (21.3)	
	≥ 3	5 (6.3)	
	Mean (SD)	0.6 (1.1)	
Dental trauma	Yes	5 (6.3)	< **0.0001** *
	No	**75 (93.8)**	
Fluorosis	Normal (DI = 0)	**75 (93.8)**	< **0.0001** **
	Questionable (DI = 1)	3 (3.8)	
	Very mild (DI = 2)	2 (2.5)	
Gingivitis	Yes	6 (7.5)	< **0.0001** *
	No	**74 (92.5)**	

Abbreviations: DMFT, decayed, missing, and filled teeth in the permanent dentition; dmft, decayed, missing, and filled teeth in the primary dentition; SD, standard deviation.

* Chi‐squared independence test.

** Chi‐squared partition test.

Table 2 shows the common and specific questions in the short‐forms. CEDAM‐ISF:8 and CEDAM‐RSF:8 have 4 questions in common: “Worry if need to have something done,” “Think they will stop if asked,” “Think sill have control over what happens” (thoughts) and “Feel trust” (physical symptoms or feelings). CEDAM‐ISF:8 also included two questions about behaviors (Items 2 and 3), one about thoughts (Item 6) and one about physical symptoms or feelings (Item 13). On the other hand, three of the last four questions of CEDAM‐RSF:8 concern about physical symptoms or feelings (Items 10–12) and one concerns about thoughts (Item 7).

**TABLE 2:** Questions in the CEDAM‐ISF:8 and CEDAM‐RSF:8.

CEDAM‐ISF:8 specific questions	Common questions	CEDAM‐RSF:8 specific questions
2. Talk to parents about whether want to go (B)	4. Worry if need to have something done (T)	7. Think things could go wrong (T)
3. Let the dentist look in mouth (B)	5. Think they will stop if asked (T)	10. Feel stressed (P/F)
6. Think it will be painful (T)	8. Think will have control over what happens (T)	11. Feel upset (P/F)
13. Feel angry (P/F)	14. Feel trust (P/F)	12. Feel embarrassed (P/F)

Abbreviations: B, behaviors; CEDAM, children's experiences of dental anxiety measure; ISF, impact short‐form; P/F, physical symptoms or feelings; RSF, regression short‐form; T, thoughts.

Table 3 shows the outcomes of the descriptive analysis. There was a higher mean for the impact version (12.4 vs. 11.4), but the difference between the two versions was not significant. Both short forms had a floor effect, and only CEDAM‐ISF:8 had a ceiling effect (1.3%).

**TABLE 3:** Descriptive statistics for the CEDAM‐ISF:8 and CEDAM‐RSF:8.

Short‐form	CEDAM‐ISF:8	CEDAM‐RSF:8
Range of possible values	8–24	8–24
Mean (standard deviation)*	12.4 (3.8)	11.4 (2.8)
Range of scores	8–24	8–21
% with score of 0	3.8	10.0
% with maximum score	1.3	0.0

Abbreviations: CEDAM, children's experiences of dental anxiety measure; ISF, impact short‐form; RSF, regression short‐form.

* *p* = 0.077 (obtained from Mann–Whitney test).

In Table 4, it was observed that there was a strong positive correlation between the scores of short forms and CEDAM (rho > 0.90; *p* < 0.001), suggesting that the formers correspond to the criteria assessed in the original version; that is, confirming the criteria validity of the short forms.

**TABLE 4:** Criterion validity: correlations between short‐forms and the long‐form of CEDAM.

Short‐form	rho^a^	*p*
CEDAM‐ISF:8	0.95	< 0.001
CEDAM‐RSF:8	0.98	< 0.001

Abbreviations: CEDAM, children's experiences of dental anxiety measure; ISF, impact short‐form; RSF, regression short‐form.

^a^ Spearman's correlation coefficient.

Table 5 shows the significant positive correlation between the scores of the two short forms and the overall CFSS‐DS score (*p* < 0.001), suggesting that the greater the dental anxiety, the greater the dental fear. The magnitude of the associations was moderate. These findings indicated satisfactory correlational construct validity of the short versions.

**TABLE 5:** Correlational construct validity: correlations between scores of the short‐forms and the CFSS‐DS.

CFSS‐DS		
Short‐form	rho^a^	*p*
CEDAM‐ISF:8	0.55	< 0.001
CEDAM‐RSF:8	0.57	< 0.001

Abbreviations: CEDAM, children's experiences of dental anxiety measure; ISF, impact short‐form; RSF, regression short‐form.

^a^ Spearman's correlation coefficient.

The discriminant construct validity was shown in Table 6, with a significant difference in the means of the short versions between the CFSS‐DS categories. These findings suggested that children with a high fearful (CFSS‐DS ≥ 32) reported greater dental anxiety compared to those classified as having low fearful (CFSS‐DS < 32).

**TABLE 6:** Discriminant construct validity: comparison in scores of the short‐forms by categories of CFSS‐DS.

CFSS‐DS	*n*	CEDAM‐ISF:8	CEDAM‐RSF:8
		Mean (SD)	Mean (SD)
Low fearful (score < 32)	29	11.0 (1.1)	10.0 (1.2)
High fearful (score ≥ 32)	51	12.0 (4.3)	11.0 (3.1)
		*p* * = 0.0001	*p* * = 0.0001

Abbreviations: CEDAM, children's experiences of dental anxiety measure; CFSS‐DS, children's fear survey schedule‐dental subscale; ISF, impact short‐form; RSF, regression short‐form.

* Mann–Whitney test.

The reliability results are shown in Table 7. Cronbach's alpha values for CEDAM‐ISF:8 (*α* = 0.90) and CEDAM‐RSF:8 (*α* = 0.80) indicate, respectively, “almost perfect” and “substantial” internal consistency reliability. There was little change in the Cronbach's alpha coefficients when individual items were deleted. The magnitude of the corrected item total correlations ranged from “moderate” to “strong” for CEDAM‐ISF:8 and from “weak” to “moderate” for CEDAM‐RSF:8.

**TABLE 7:** Reliability statistics: short‐forms of the CEDAM.

Short‐form	CEDAM‐ISF:8	CEDAM‐RSF:8
Cronbach's alpha	0.90	0.80
Range of alpha's if items deleted	0.87–0.90	0.76–0.81
Range of corrected item total correlations	0.52–0.78	0.24–0.66

Abbreviations: CEDAM, children's experiences of dental anxiety measure; ISF, impact short‐form; RSF, regression short‐form.

## 4 Discussion

Short‐forms can expand the utilization of a questionnaire, reducing the time, the costs associated with evaluation and the proportion of missing items [10, 27]. In clinical practice, brief scales are recommended as clinicians lack time and expertise in administering anxiety scales [8]. In addition, brief and straightforward measures that require no additional scoring [8] can be advantageous for measuring children's dental anxiety in routine assessments [13]. In this study, the item impact and regression methods were used to create the short‐forms of the Brazilian CEDAM, considering a theoretical model on which the original version was based. The first selects item that are the most important to patients, while in the latter, items for the short‐form are selected based on their ability to predict overall scale score [16]. There is no superiority in the different methods, item impact and regression, but the content and properties of the respective measures seem to be relevant [17]. Each of the shortening techniques produced an 8‐item measure, that is, CEDAM‐ISF:8 and CEDAM‐RSF:8, which had four items in common; three concern “thoughts” and one considers “physical symptoms or feelings.” Porritt et al. [13] also found four common items using impact and regression methods, 2 assessing “thoughts,” and 2 assessing “physical symptoms or feelings”. Only item 4 (“feeling worried about the need to have something done”) was similar to the common items observed in the present study.

The presence of floor or ceiling effects registers content validity, with minimum/maximum scores below 15% being considered acceptable [30]. In this study, 3.8% and 10% of children had zero scores on the CEDAM‐ISF:8 and CEDAM‐RSF:8, respectively. The latter did not show ceiling‐effects, while they were minimal for the CEDAM‐ISF:8 questionnaire (1.3%). Similarly, floor (3.5%) and (1.2%) ceiling effects were almost non‐existent for the English CEDAM‐8, using data from a sample of 99 nine‐ to sixteen‐year‐old children, recruited from a United Kingdom Pediatric Dental Hospital [13]. On average, the item‐impact version of the Brazilian CEDAM seems to detect higher levels of dental anxiety than the regression ones (12.4 vs. 11.4, *p* = 0.077), probably due to the selection of the items with the highest impact (score 3).

Each of the shortforms developed has also been tested for validity and reliability. Criterion validity is tested by comparing the questionnaire with other measures considered to be standard external criteria. It was observed that significant correlations existed between the 14‐item CEDAM and the short forms, indicating that they are evaluating the same concept. Despite the correlation coefficients for the short forms being nearly identical, the association was somewhat stronger for the CEDAM‐RSF:8 (rho = 0.98) in comparison to CEDAM‐ISF:8 (rho = 0.95), probably due to the selection of items that explain the most variation in the total CEDAM score. In the Porritt study [13], a correlation coefficient of 0.90 was found as a criterion validity of the English CEDAM‐8. However, these authors used a mixture of the four common questions and the two highest‐ranking questions from each method to develop a unique CEDAM‐8. Considering the particularity of each approach, resulting in specific short forms that differ in items and measurement properties [27], the comparison between the studies should be done with caution.

The content validity of a questionnaire is influenced by the reduction of the number of items [27]. While the content relevance is the same, the content coverage is reduced [27], potentially reducing the construct validity. The present outcomes showed good correlational construct validity, with moderate correlation between the overall CFSS‐DS score and the CEDAM‐ISF:8 (rho = 0.55) and the CEDAM‐RSF:8 (rho = 0.57). These values were almost identical to the correlation found for the long‐form of the CEDAM (rho = 0.56) and CFSS‐DS scores (data not published), suggesting moderate agreement between dental anxiety and dental fear scales. These findings could be explained by the clear difference between fear of a dentist and the fearful behavior exhibited by children, because the behavior can be a result of their dental fear and their ability to deal with invasive situations [25].

The concept of discriminant validity is an important part of the measure's construct validation process and refers to the ability of the proposed measure not to be modified by processes that are theoretically unrelated to the object of the questionnaire [31]. In the present research, discriminant construct validity was tested by comparing the means of the short forms across the categories of the CFSS‐DS. Both short forms recognized variances in dental anxiety between the two groups in a predictable way; that is, the scores were significantly higher in the children with high fear (*n* = 51, CFSS‐DS ≥ 32) when compared with their counterparts (*n* = 29, CFSS‐DS < 32).

Internal consistency was determined by the Cronbach's alpha coefficient, which assesses whether the degree of total variance in the test results is associated with the sum of the variance from item to item [32]. CEDAM‐ISF:8 and CEDAM‐RSF:8 had satisfactory Cronbach's alpha values, indicating “almost perfect” (*α* = 0.90) and “substantial” (*α* = 0.80) internal consistency, respectively [29]. Despite the alpha being lower than those observed for long‐form CEDAM (*α* = 0.93) (data not published), both were above the acceptable value (0.60) estimated for questionnaires with a reduced number of items [33]. However, CEDAM‐RSF:8 (*α* = 0.80) suggests possible limitations for individual‐level assessments since they require that reliability coefficients are at least 0.90 [34, 35].

Overall, the present study shows evidence about psychometric properties of the CEDAM‐ISF:8 and CEDAM‐RSF:8. However, considering the reliability results, more stable results were observed for the item impact than the regression method. The selection of the most relevant items to the respondents of the questionnaire is an advantage of the item impact approach, considering that they are the main experts about dental anxiety. A limitation of the present study is the use of data collected in a validation study for the original questionnaire; thus, further research using the short forms administered on their own is needed to reinforce their measurement properties. Moreover, the cross‐sectional properties of the measures need further testing in replicated studies involving longitudinal design, clinical samples, and different settings to ensure other measurement properties, such as responsiveness to change. Future research should also focus on exploring the experience and perceptions of dentists regarding child's anxiety evaluation to know and to control potential barriers that are likely to prevent clinicians from undertaking patient‐centered assessments in routine dental care.

## Author Contributions

L.D.S.F. analyzed and interpreted the data; and drafted the manuscript. J.H.L.S. acquired the data and critically revised the manuscript. B.S.R. interpreted the data and critically revised the manuscript. T.S.B. conceived and designed the study; acquired, analyzed, and interpreted the data; drafted the manuscript; and critically revised the manuscript. All authors have read and approved the final manuscript.

## Conflicts of Interest

The authors declare no conflicts of interest.

## Data Availability

The data of this study are available from the corresponding author upon reasonable request.
